# Study of the spatial connection between urbanization and the ecosystem—A case study of Central Yunnan (China)

**DOI:** 10.1371/journal.pone.0238192

**Published:** 2020-09-29

**Authors:** Yuqin Yang, Zhang Jun, Xin Sui, Xiong He

**Affiliations:** School of Architecture and Planning, Yunnan University, Yunnan, China; Northeastern University (Shenyang China), CHINA

## Abstract

This study provides new perspectives on urban development and conservation by exploring the spatial interaction between ecosystem services and urbanization. Limited studies have discussed the interaction between ecosystem services and urbanization; therefore, in this research, the spatial relationship between ecosystem services and urbanization is explored by taking the urban agglomeration in Central Yunnan as an example, and land-use data and economic and social data from 2009 and 2018 are used to determine the interactive impact of urbanization on the urban ecosystem. It is shown that (1) the spatial distribution of the urbanization level of the urban agglomeration in Central Yunnan has significant regional differences, showing a decreasing trend from the urbanized area to the surrounding areas. (2) Another factor with obvious regional differences is the spatial distribution of ecosystem services, which is similar to urbanization in spatial distribution. This difference is mainly caused by the impact of the urbanization level and the change in land use. (3) The spatial distribution and local agglomeration of urbanization and the ecosystem service value of urban agglomeration in Central Yunnan are very similar, and there is a significant negative correlation between the urbanization level and ecosystem service value. The research results have guiding significance for future urban and ecological development in Central Yunnan city.

## 1. Introduction

The development of urbanization has led to many changes. On the one hand, urbanization drives the overall development of the economy, culture and society of the city. On the other hand, human activities related to urbanization are changing the structure and processes of the ecosystem [1], including vegetation coverage [2], land use [3] and species reduction [4], which results in the transformation of an ecosystem dominated by nature into an ecosystem dominated by human beings or the coupled domination of human beings and nature. At present, the ecosystem is suffering from great pressure due to urbanization. Understanding how to reduce the impact of urbanization on the ecosystem while realizing rapid urbanization and urban sustainable development has become a hot topic considered by scholars and decision makers in urban, ecological and other fields around the world [5–7].

Ecosystem services (ESs) refer to the commodities and services directly or indirectly obtained by humans from the ecosystem and represent the link between the ecosystem and human welfare [8]. ESs can be divided into four categories: supply services (food production, raw material production, etc.), regulatory services (climate regulation, gas regulation, etc.), support services (biodiversity maintenance, soil protection, etc.) and cultural services (outdoor entertainment, aesthetic landscapes, etc.) [9–11]. Costanza quantifies ecosystem service value (ESV) and presents it in the form of currency [12], providing theoretical support for later research on the ESV [13].

The ESV quantifies the functions of ecological services, making ESV an important indicator for ecological evaluations [14]. Due to the degradation of ecosystem functions, understanding how urbanization affects the ESV has become an important research topic in ecology, geography and other disciplines [15,16]. Rapport developed a comprehensive framework for environmental statistics called the pressure-state-response model (PSR), which has been widely used in the evaluation of urban ecological health [17]. Odum and others analysed the impact of urban development and environmental evolution in typical urbanized areas [18] by using system dynamics and sensitivity models.

Urbanization mainly refers to the expansion of urban built-up areas [19] that changes the process and structure of the existing ecosystem and greatly affects the ecosystem's ability to provide services to people [20,21]. Many prior studies have shown that the transformation of various types of land to built-up areas will lead to a decrease in the ESV to different degrees [22,23]. In the current process of urbanization, most of the impacts of land-use transformation on ESs represent one way to calculate the role of ESV in urbanization from an economic perspective [24–26], while less consideration is given to the spatial linkage generated by changes in the supply of and demand for urbanization and ESV [27,28].

In contrast to other regions, the ESs of urbanized regions are unique and scarce due to population agglomeration and economic development [29,30]. When such special uniqueness and scarcity change due to the supply of and demand for ESs, the total value of ESs in urbanized areas will fluctuate [31], and the ESV may increase or decrease [32]. This topic has been ignored in previous studies. Therefore, when objectively measuring the total ESV, it is necessary to consider not only the calculation of ESV for different land types [33,34] but also the reverse impact of urbanization on ESV [35].

In recent decades, China's cities have experienced unprecedented rapid expansion, and the urbanization rate exceeds 60% [36], which indicates that China's urbanization has maintained the momentum of rapid and sustained growth. This study objectively calculates the value provided by the ecosystem in hopes that the spatial relationship between the degree of urbanization and the ESV can be revealed to help relevant decision makers understand the importance of this relationship for future urban construction and development.

As one of the most important examples of urban agglomeration in western China, the urban agglomeration in Central Yunnan has led to the problem that the encroachment on ecological space and resources and environment has increasingly intensified, as has the demand for ESV, due to the rapid development of urbanization [37]. In this study, urban agglomeration in Central Yunnan is selected as the research object, and data on land use, economy and society are selected to quantify and map ESV and urbanization; a spatial statistical model is used to analyse the spatial relationship between ESV and urbanization. The goal is to reveal the characteristics of the spatial interaction and agglomeration and to provide a theoretical basis for the sustainable development planning of urban agglomeration.

## 2. Study area

**Fig 1:**
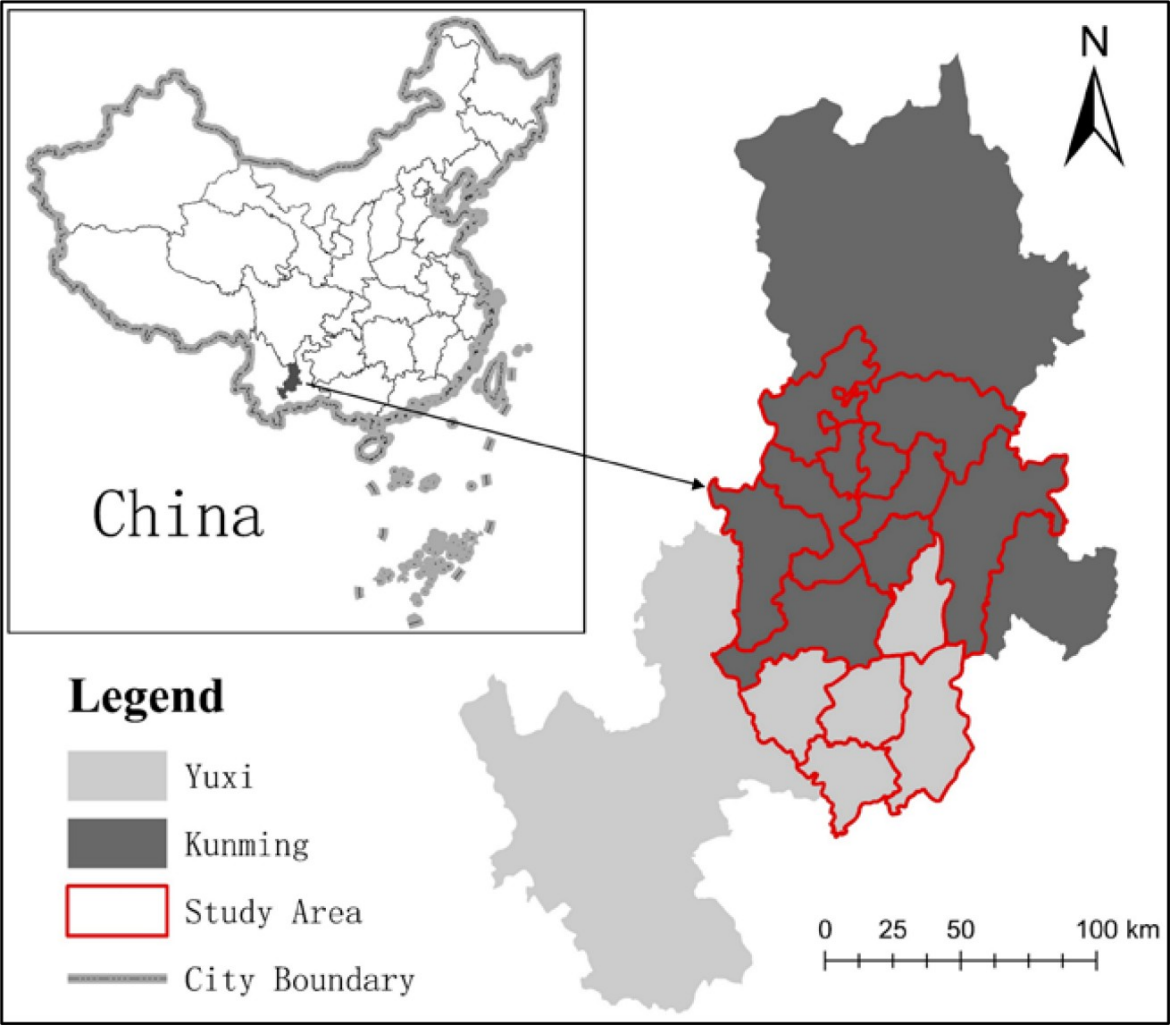
Study area. (Source of base map: the open source map data service provided by the National Platform for Common GeoSpatial Information Services (https://www.tianditu.gov.cn/)).

This study was conducted in Yunnan, China (23'54'25', 25'61'08; 102'11'36, 103'28'03). This study focuses on the urban agglomeration of Central Yunnan, which is in southwest China, and its total area is 141 square hectares (Fig 1). This area has high research value because it contains not only a rapidly urbanized main city area and radiation-driven area but also five great lakes and ecological areas with high forest coverage [37]. At the macro level, the research area is positively influenced by the policies of the central government, such as the "One Belt and One Road" initiative and "Yangtze River Economic Belt". At the micro level, this area is positively influenced by local government policies, such as “the framework agreement on promoting the integrated development and cooperation of KunYu in Central Yunnan urban agglomeration, Kunming master plan (2018–2035)”. In recent years, due to the significant increase in the level of urbanization in the study area, the ecological environmental problems caused by rapid urbanization have become increasingly serious, and the ecological system has been seriously damaged due to the drastic expansion of urban agglomeration in Central Yunnan. Therefore, an analysis of the objective connection between urbanization and ecological service systems is urgently needed.

## 3. Methodology

### 3.1 The sources and processing of the data

This study mainly uses land-use data and economic and social data. Land-use data were obtained through the Operational Land Imager + Thermal Infrared Sensor (OLI+TIRS) (http://www.gscloud.cn/) and images obtained by Landsat 8, while social and economic data were obtained from the statistical yearbooks of Yunnan Province for 2009 and 2018 (http://stats.yn.gov.cn/tjsj/tjnj/), the compilation of cost and income data on national agricultural products (http://www.shujuku.org/agr-products-cost-benefit.html), and pollution charge collection methods and standards.

### 3.2 Research methods

#### (1) Calculation of ESV

In this paper, a table of the individual ESV coefficients (Table 1) of different land types per unit area in the Central Yunnan urban agglomeration area is established by using the ESV equivalent of the Chinese ecosystem unit area formulated by Xie Gao-di and others [38], showing the differences between regions and the ESV correction parameters (1.95) of the urban agglomeration in Central Yunnan. The ESV of the research area is calculated based on the information provided in Table 1. The value of the ESV-equivalent factor is calculated as follows:

$${ESV}_{i}=\sum_{j}^{m}A_{j}\times {VC}_{ij}$$ (1)

$${TV}_{j}={VC}_{ij}\times A_{j}$$ (2)

$$ESV=\sum_{i}^{n}{ESV}_{i}=\sum_{j}^{m}{TV}_{j}$$ (3)

where *ESV*_*i*_ stands for the ecological service function value of item i; TV_j_ represents the ecological service value of type j land; ESV represents the total value of ecological services; and VC_ij_ represents the unit price of the ecological service function of I of the type j land. A_j_ represents the land area of the type j land; m represents the category of land use; n represents the category of ecological service function.

**Table 1:** ESV coefficients per unit area of land-use types (hm^−2^ a^−1^).

ES function	Forestland	Grassland	Cultivated land	Water body	Unused land	Construction land
Food production	607.76	791.93	1841.69	976.09	36.83	0.00
Raw materials	5488.23	663.01	718.26	644.59	73.67	0.00
Gas regulation	7956.09	2762.53	1326.01	939.26	110.50	-1113.38
Climate regulation	7495.66	2873.03	1786.44	3793.87	239.42	0.00
Water conservation	7532.50	2799.36	1418.10	34568.45	128.92	-11190.01
Waste treatment	3167.70	2431.03	2559.94	27349.04	478.84	-1773.55
Soil formation and protection	7403.58	4125.38	2707.28	755.09	313.09	0.00
Biodiversity protection	8306.01	3443.95	1878.52	6316.98	736.67	0.00
Recreational culture	3830.71	1602.27	313.09	8177.09	442.00	442.00
Total	51788.22	21492.48	14549.32	83520.48	2559.94	-13634.94

#### (2) Evaluation of urbanization

Urbanization can be comprehensively evaluated from four aspects: population urbanization, economic urbanization, social urbanization and spatial urbanization. The population and economy are the basis of urbanization, while society and space are the manifestations of urbanization. This research measures population urbanization by the proportion of non-agricultural workers in the total population and population density while considering the natural environment. Economic urbanization is measured by the per capita GDP; social urbanization is measured by retail sales of social consumer goods; and spatial urbanization is measured by the proportion of construction land. The comprehensive urbanization level (CUL) standardizes the four indicators into a unified comprehensive index, and the standardization formula is as follows:

$$U_{i,j}^{\prime }=\frac{U_{i,j}-U_{i,\min}}{U_{i,max}-U_{i,\sin}}$$ (4)

where *U*_*i*,*j*_ stands for the original value of the urbanization index of letter i in the j grid. $U_{i,j}^{\prime }$ represents the standardized value of *U*_*i*,*j*_; and *U*_*i*,*max*_ and *U*_*i*,min_ represent the minimum and maximum values of the urbanization index of Number I in all grids, respectively.

#### (3) Spatial correlation analysis of ESs and urbanization

The Getis-Ord Gi* index is used to measure the overall and local characteristics, structural patterns and spatial clustering of the urban space [39]:

$$Z(G_{i}^{*})=\sum_{j}^{n}w_{ij}(d)x_{j}∕\sum_{j}^{n}x_{j}$$ (5)

where *w*_*ij*_(*d*) represents the spatial weight of the research area and *x*_*i*_ and *x*_*j*_ represent the index of expansion intensity of urban land use.

## 4. Results and discussion

### 4.1 Spatial distribution pattern of ESV

The total ESV for 2009 and 2018 is 39.423 billion yuan and 37.130 billion yuan, respectively (Table 2). The ESV has decreased by 2.293 billion yuan in the last 10 years. In 2009, the different land types were forestland, water area, cultivated land, unused land and construction land, provided here in the order of ESV from largest to smallest. In 2018, the order changed to forestland, water area, unused land, cultivated land and construction land. Although the change in the ESV of the other four land types is within a reasonable range, which has little impact on the total ESV in the study area, the ESV of cultivated land reduced from 2.089 billion yuan to 726 million yuan, and the ESV of construction land increased from -1.538 billion yuan to -2.108 billion yuan. The value of the increase and decrease of these two land types is nearly one-half the original values.

**Table 2:** Summary of the value of various ess in the research area in 2009 and 2018.

ESV	Forestland	Grass land	Cultivated land	Water area	Unused land	Construction land	Total
Food production	3.4	1.32	2.64	0.62	0.14	0	8.13
Raw material production	30.75	1.11	1.03	0.41	0.27	0	33.57
Gas regulation	44.57	4.61	1.9	0.6	0.41	-1.26	50.84
Climate regulation	41.99	4.8	2.56	2.42	0.88	0	52.66
Hydrologic regulation	42.2	4.67	2.04	22.05	0.47	-12.62	58.82
Waste treatment	17.75	4.06	3.68	17.45	1.76	-2	42.69
Soil maintenance	41.48	6.89	3.89	0.48	1.15	0	53.88
Maintaining biodiversity	46.53	5.75	2.7	4.03	2.71	0	61.72
Providing aesthetic landscape	21.46	2.68	0.45	5.22	1.63	0.5	31.93
Total	290.13	35.88	20.89	53.29	9.41	-15.38	394.23
Food production (2019)	3.35	1.1	0.92	0.68	0.17	0	6.22
Raw material production (2019)	30.26	0.92	0.36	0.45	0.33	0	32.32
Gas regulation (2019)	43.87	3.84	0.66	0.65	0.5	-1.72	47.8
Climate regulation (2019)	41.33	3.99	0.89	2.64	1.08	0	49.94
Hydrologic regulation (2019)	41.53	3.89	0.71	24.07	0.58	-17.3	53.48
Waste treatment (2019)	17.47	3.38	1.28	19.04	2.16	-2.74	40.58
Soil maintenance (2019)	40.82	5.73	1.35	0.53	1.41	0	49.85
Maintaining biodiversity (2019)	45.8	4.79	0.94	4.4	3.32	0	59.24
Providing aesthetic landscape (2019)	21.12	2.23	0.16	5.69	1.99	0.68	31.87
Total (2019)	285.56	29.87	7.26	58.15	11.53	-21.08	371.3

From the perspective of the structure of each single ESV, maintaining biodiversity is the dominant ES function of urban agglomeration in Central Yunnan, accounting for more than 15% in both 2009 and 2018. Next are the hydrologic regulation and soil maintenance functions, with a proportion over 13%. Gas regulation and climate regulation are the next two functions that deal with waste treatment and raw material production, among which gas regulation and raw material production are relatively stable, accounting for approximately 12% and 8%, respectively. Food production has the lowest of all ESVs, accounting for only 1.67% in 2018. Thus, it can be concluded that the ranking of the proportion of various ES functions in different periods vary little, mainly fluctuating up or down one place in the raking, and the value composition of each ES function is relatively stable.

**Fig 2:**
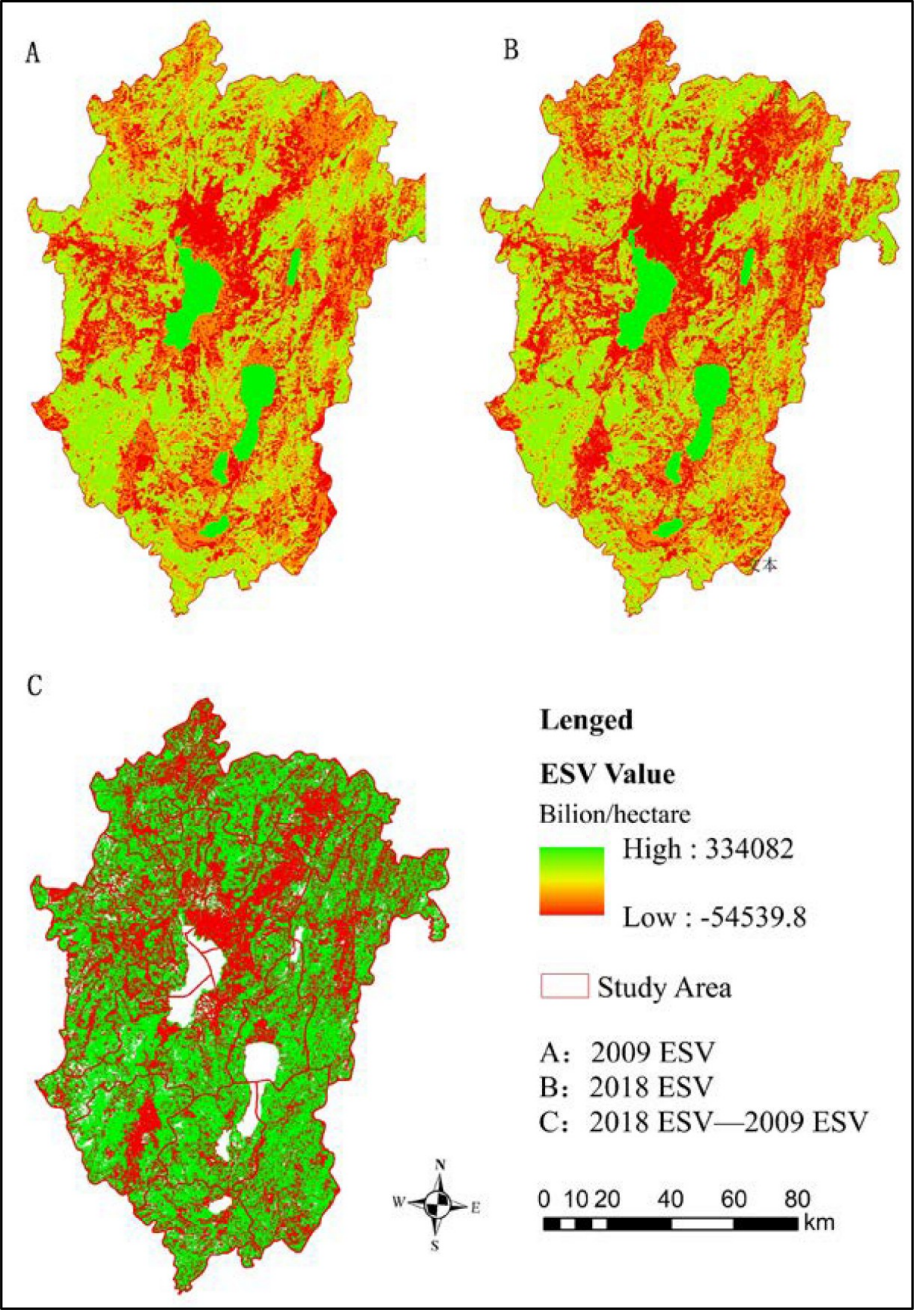
Incremental spatial distribution of the ESVs in 2009 and 2018. (Source of base map: The open source map data service provided by the National Platform for Common GeoSpatial Information Services (https://www.tianditu.gov.cn/)).

A review of the spatial distribution of the ESVs (Fig 2) indicate obvious differences in their levels, and the distribution of high and low values is more balanced in the whole research area; Dianchi Lake and Fuxian Lake are the two core circles in the high-value area. The spatial distribution of ESVs in 2009 is generally similar to that in 2018. The high ESV in 2009 is mainly due to the increase in forest area due to the long-term implementation of policies such as "afforestation" and "developing the district with ecology". The main reason for an annual expansion in the low-value area in 2019 is the rapid development of the city, which changed the land-use patterns of grassland, cultivated land and unused land, resulting in the continuous reduction in the ESVs and the diffusion of the scope.

Overall, in the 10-year period from 2009 to 2018, the change in the value is widely distributed in the region, covering almost the entire area of urban agglomeration. The distribution of the areas with increased value is relatively scattered and fragmented; these areas are mainly concentrated in the main urban areas of Kunming and Yuxi. This may have occurred due to policies with various areas of focus, such as "return the grain plots to forestry", "wetland protection", "afforestation" and " developing the district with ecology". On the other hand, in contrast to the areas with increased values, the areas with declining values are relatively concentrated and distributed, mainly along the southeastern coast of north Dianchi Lake. These areas combine new and old urban areas and the area along the airport expressway, which is highly affected by the construction of new cities, the transformation of farmland to other high-efficiency economic production land, and the construction of transportation infrastructure. Due to changes in the urbanization level and land use, the spatial distribution of ESs has obviously changed.

### 4.2 Spatial distribution pattern of urbanization

Using the method to evaluate urbanization discussed in chapter 3, an index for population urbanization, economic urbanization, social urbanization, spatial urbanization and comprehensive urbanization index can be calculated, as shown in Table 3.

**Table 3:** Urbanization level of urban agglomeration in Central Yunnan.

	Comprehensive urbanization	Population urbanization	Economic urbanization	Social urbanization	Spatial urbanization
2009	4.4956	0.8513	2.6188	0.4417	0.5838
2018	5.3698	1.0081	3.1509	0.6026	0.6082
Rate of change	34.62%	26.40%	40.51%	52.69%	9.77%

As shown in Table 3, in order of the proportion contributed to overall urbanization from high to low, the different aspect are economic urbanization, population urbanization, spatial urbanization and social urbanization. However, in terms of the change in each aspect of the urbanization index from 2009 to 2018, the order from high to low is social urbanization, economic urbanization, population urbanization and spatial urbanization. The level of comprehensive urbanization increased from 4.4956 in 2009 to 5.3698 in 2018, an increase of 34.62%. The urbanization caused by the population, economy, society and space has been increasing year by year, and the increase in the social urbanization rate is the most prominent, indicating that the level of living and social consumption of residents has greatly improved. The rate of increase in spatial urbanization is low compared to that of the other aspects of urbanization, and the period of spatial development is relatively slow and long. In general, the urbanization level of agglomeration of Central Yunnan has been significantly improved.

In the figure that shows the urbanization level of the urban agglomeration of Central Yunnan, the region with the highest urbanization level is Kunming, followed by Yuxi, and the counties with the lowest urbanization level are Fumin County and Huaning County. The medium level of urbanization is mainly distributed in the outer edge of the core circle of the city, concentrated in Chenggong District and Jinning District. In the past 10 years, the spatial distribution of the urbanization level in the study area has not changed much, and the urbanization level in each region is increasing. The Chenggong District of Kunming shows the most prominent rate of increase, at 236.28%. This area is close to the Kunming economic and technological development zone and Chenggong New City. Next, is Jinning district, with a rate of increase of 158.23%, which may have occurred because Jinning district is an important node of the Kunming and Yuxi economic corridors. However, the urbanization level in northern Kunming decreased slightly over 10 years, highlighting the prominent contradiction of the local market industrial structure. The spatial distribution of the urbanization level of the urban agglomeration in Central Yunnan differs significantly, and from high to low is the urbanization area, the urbanizing area, showing a decreasing trend from the urbanization area to the surrounding area.

### 4.3 Spatial correlation between urbanization and ESs

**Fig 3:**
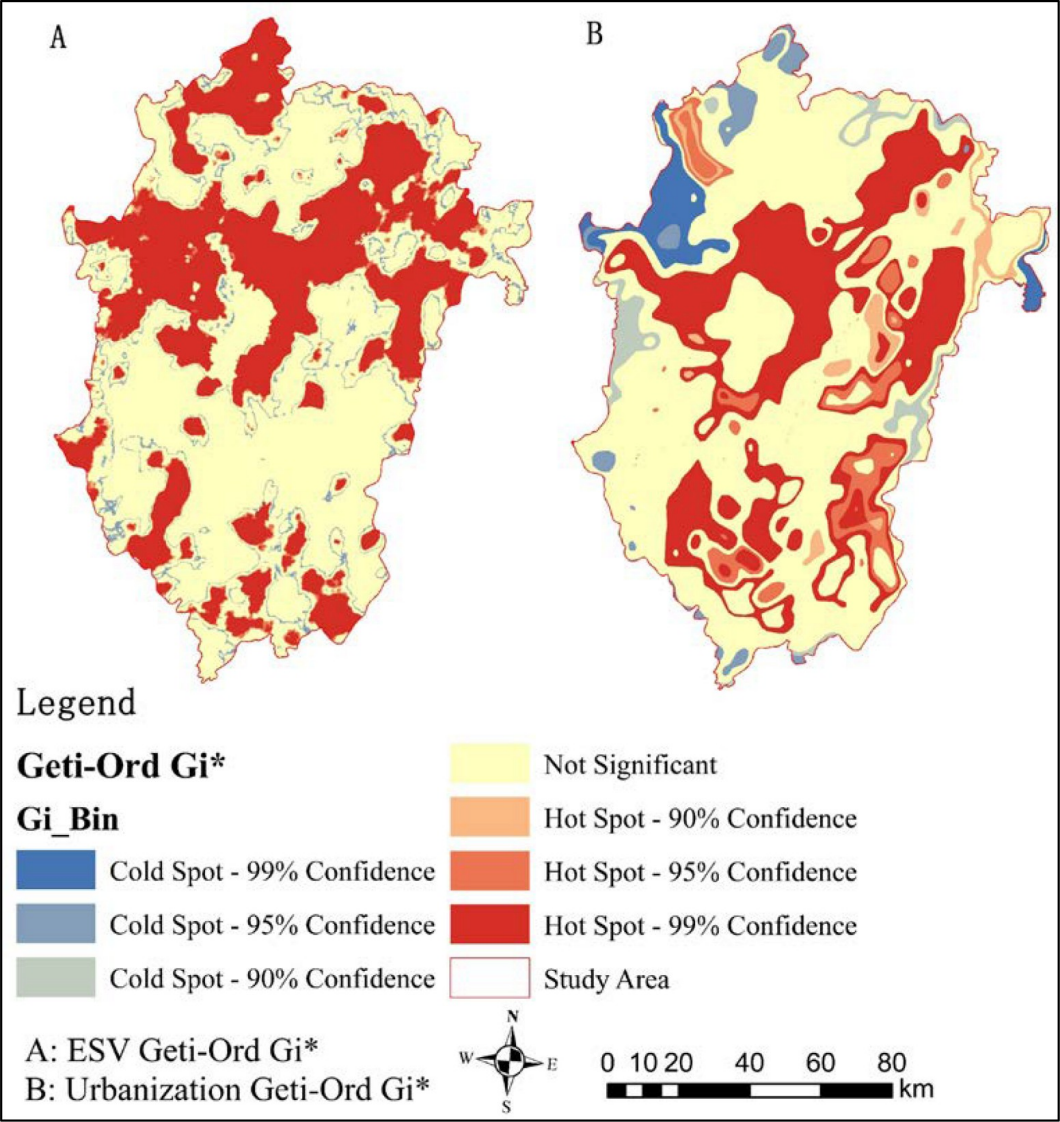
Analysis of the pattern evolution of hot spots of esv and urbanization growth from 2009 to 2018. (Source of base map: The open source map data service provided by the National Platform for Common GeoSpatial Information Services (https://www.tianditu.gov.cn/)).

The Getis-Ord Gi* in Fig 3 is obtained by analysing the figure showing the changes in ESV and urbanization from 2009 to 2018; this figure represents the correlation between ESV and urbanization across geographical locations.

**Fig 4:**
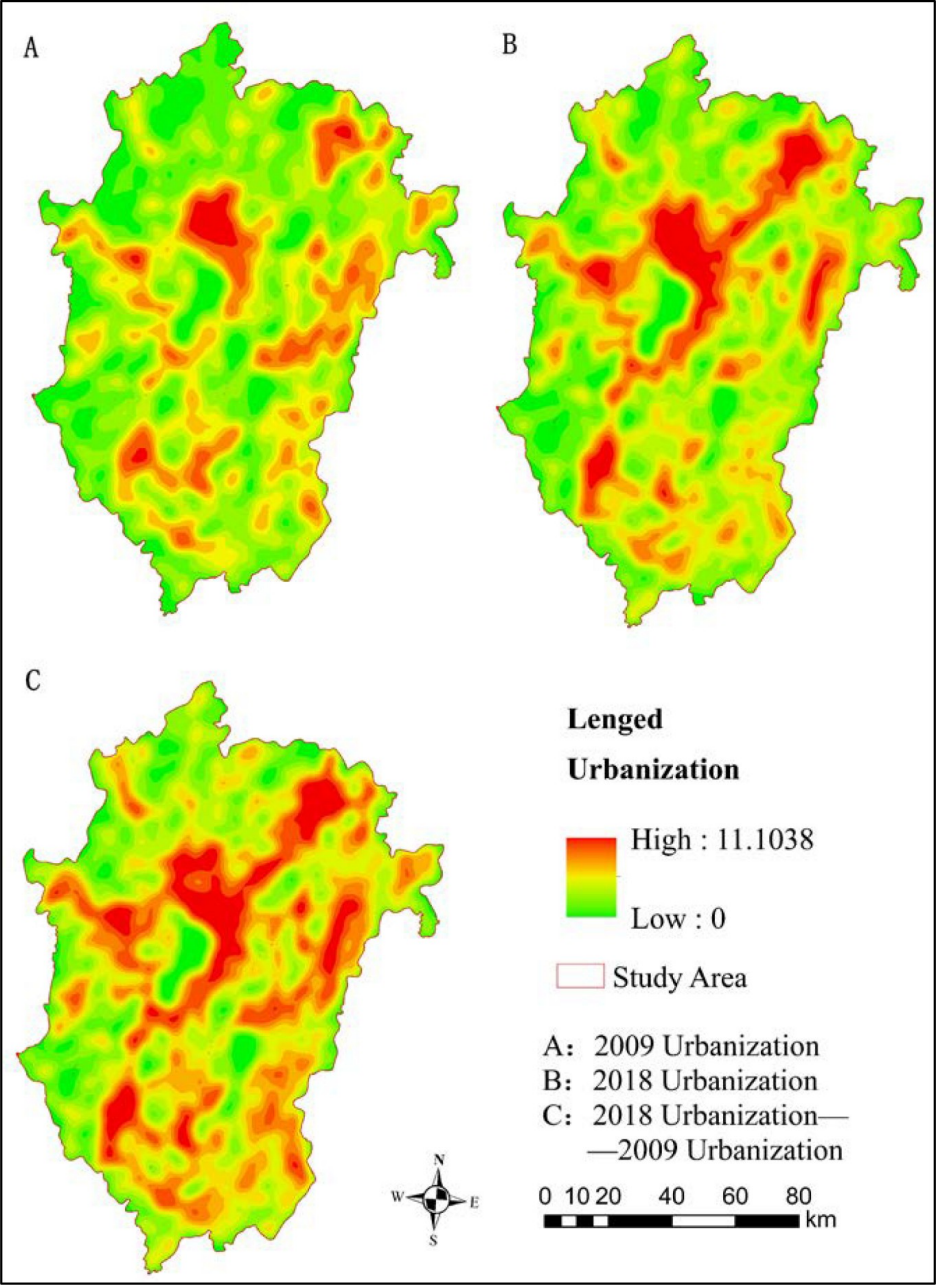
Urbanization level of urban agglomeration. (Source of base map: The open source map data service provided by the National Platform for Common GeoSpatial Information Services (https://www.tianditu.gov.cn/)).

To determine the pattern evolution of "hot spots", Getis-Ord Gi * is used to compare and analyse the spatial growth trend of ESV and urbanization from 2009 to 2018. ArcGIS is used to create the distribution map of ESV and urbanization "Hot Spot" and "Cold Spot" areas related to urban agglomeration in Central Yunnan (Fig 4). This map clearly reflects the evolution process and connection between ESV and urbanization. The evolution of the spatial pattern is divided into three categories: "Hot Spot", " Cold Spot " and "Not Significant". The "Hot Spot" area with ESV changes accounts for 32.19% of the total area, the "Cold Spot " area accounts for 65.13% of the total area, and the area indicated as "Not Significant" accounts for 2.68%. The "Hot Spot" area with changes in urbanization accounts for 30.28% of the total area, the "Cold Spot" area accounts for 61.25% of the total area, and "Not Significant" accounts for 8.47%. The scale shows that the distribution of "Hot Spot" and "Cold Spot" areas are roughly the same, but there are more "Cold Spot" areas with changes in urbanization. In general, the "Hot Spot" area is concentrated north of the urban agglomeration, and the "Cold Spot" area is concentrated south of the urban agglomeration in Central Yunnan. The change in ESV is highly consistent with the change in urbanization, which again proves the strong correlation between the two.

Both "Cold Spot" and "Hot Spot" areas show changes in the ESV and urbanization in the past 10 years. The ESV of the "Hot Spot" area accounts for 18.27% of the total ESV, while urbanization of the "Hot Spot" area accounts for 84.90% of total urbanization. In addition to changes in the "Hot Spot" area, the changes in the "Cold Spot" and "Not Significant" areas are not obvious, indicating that the ESV has an obvious negative correlation with urbanization.

Considering the calculated ESV and urbanization level index, SPSS is used to analyse the Pearson correlation between the ESV and each aspect of urbanization included in the urbanization index. The results are provided in Table 4.

**Table 4:** Correlation coefficient matrix for the urbanization level and ESV.

	Population urbanization	Economic urbanization	Social urbanization	Spatial urbanization	Comprehensive urbanization
Food production	-0.695**	-0.569**	-0.612**	-0.653**	-0.659**
Raw material production	-0.469**	-0.280	-0.421**	-0.577**	-0.409**
Gas regulation	-0.498**	-0.320*	-0.448**	-0.609**	-0.446**
Climate regulation	-0.528**	-0.345*	-0.470**	-0.617**	-0.471**
Hydrologic regulation	-0.618**	-0.525**	-0.541**	-0.679**	-0.607**
Waste treatment	-0.578**	-0.519**	-0.488**	-0.537**	-0.551**
Soil maintenance	-0.524**	-0.350*	-0.469**	-0.611**	-0.472**
Maintaining biodiversity	-0.552**	-0.372*	-0.490**	-0.633**	-0.497**
Providing aesthetic landscape	-0.585**	-0.404**	-0.508**	-0.637**	-0.525**
Total value of function	-0.603**	-0.433**	-0.532**	-0.678**	-0.554**

** indicates a significant correlation at the 0.01 level

* represents a significant correlation at the 0.05 level.

The table shows that the CUL has a negative correlation with the total ESV, with a correlation coefficient of -0.554, indicating that the higher the degree of urbanization, the lower the ESV. In addition, the relationship between the four dimensions of urbanization and different ES functions is inconsistent. The negative correlation of spatial urbanization is the strongest, with a correlation coefficient of -0.678, followed by population urbanization, with a correlation coefficient of -0.603, indicating that the more obvious the expansion of urban construction and the more densely populated the area, the lower the service value. By comparing Fig 2 and Fig 4, it becomes obvious that regions with higher urbanization levels have lower ESVs. In recent years, due to the rapid development of urbanization in Kunming and Yuxi, the total ESVs in the whole research area have declined.

The correlations between the level of urbanization and the service value of different ecosystems differ. Urbanization has a strong negative correlation with food production, hydrological regulation and waste treatment, and the correlation coefficients are -0.659, - 0.607, and -0.551, respectively. The main reason for this result is that certain land-use types, such as cultivated land and grassland, have been transformed to residential building land, traffic road construction land and industrial and mining land. Due to the modern development of the city, the decrease in cultivated grassland area is leading to a decline in food production and other functions. At the same time, Due to the expansion of the urban scale and the continuous increase in urban construction land, many types of pollution are discharged into urban areas as well as rivers, lakes and other waters one after another. Some toxic waste will even infiltrate into soil, which will cause serious pollution and eutrophication of the water area, destroy the structure of the water ecosystem, and lead to a decline in the functional value of hydrological regulation, waste treatment and so on.

## 5 Discussion and conclusion

The rapid development of urbanization has brought about a great change in the ESV system dominated by nature, and urbanization has had an important negative impact on the ecosystem. This study mainly analyses the correlation between the urbanization level and the ESV and concludes that a negative correlation exists; that is, the higher the urbanization level is, the lower the ESV.

The change in the ecosystem caused by an increase in the urbanization rate affects the ecosystem service function. The main urban areas of Kunming and Yuxi, which have the highest urbanization levels, are densely populated and have developed industry and commerce, have a low ESV. In this study, the relation between the ESV and urbanization is not only evaluated from the perspective of economic quantity, but the unique role of urbanization in ESs is considered. This study considers the effect of the fluctuation in the ESV caused by this uniqueness and the negative impact of hydrology and waste utilization on the ESV, as well as the spatial relationship and changes in the two-way supply and demand relationship between the ESV and urbanization. It is helpful to understand the ESV, the spatial clustering distribution and correlation between the ESV and urbanization.

In this study, in the time series, the ESV of the whole research area does not show a downward trend with increasing urbanization year by year, and more attention should be paid to this phenomenon. There is a possibility that the relationship between the ESV and urbanization is in a moderate state of coordination; that is, as the urbanization level increases, the ESV also increases. It would be worthwhile to increase the data in the time series to better explore this. By analysing the relationship between the ESV and urbanization, this study can act as a reference for subsequent studies on ESs in urban agglomerations.

The ESVs for 2009 and 2018 are calculated by using the equivalent factor method proposed by Xie Gao-di, and the urbanization levels of 2009 and 2018 are calculated by considering population urbanization, economic urbanization, social urbanization and spatial urbanization. In addition, a spatial comparison and correlation coefficient analysis are carried out. The following conclusions are drawn.

Due to the differences in development factors such as location, ecological environment and regional policy, the spatial distribution of the urbanization level of the urban agglomeration in Central Yunnan is significantly different. The spatial pattern shows a decreasing trend from the urbanization area to the surrounding area. The urbanized area has the highest level of urbanization and that of the urbanizing area is lower than that of the urbanized area. The ecological core area has the lowest level of urbanization.

The spatial distribution of ESs is obviously different due to the influence of urbanization and land use changes. The spatial pattern is distributed along the water system and traffic route and shows an outward-increasing spatial distribution trend. Low-value ESs are mainly distributed in urbanized areas, while high-value ESs are mainly concentrated in urbanizing areas, water systems and forest areas.

There is a negative correlation between the level of urbanization and the ESVs, indicating that the higher the degree of urbanization is, the lower the value of services. Spatial urbanization has the strongest negative correlation with the ESV, followed by population urbanization, indicating that the more obvious the expansion of construction land is, the more densely populated the region is, and the lower the ESV will be. Moreover, the ESs of local agglomeration and urbanization are very similar.

In other words, although urbanization has a negative impact on ESs to some extent, reasonable urban planning and policy guidance can avoid the decline of the function of urban ESs and even improve ESs.
